# Neural Infection by Oropouche Virus in Adult Human Brain Slices Induces an Inflammatory and Toxic Response

**DOI:** 10.3389/fnins.2021.674576

**Published:** 2021-11-23

**Authors:** Glaucia M. Almeida, Juliano P. Souza, Niele D. Mendes, Marjorie C. Pontelli, Nathalia R. Pinheiro, Giovanna O. Nogueira, Ricardo S. Cardoso, Isadora M. Paiva, Gustavo D. Ferrari, Flávio P. Veras, Fernando Q. Cunha, Jose A. C. Horta-Junior, Luciane C. Alberici, Thiago M. Cunha, Guilherme G. Podolsky-Gondim, Luciano Neder, Eurico Arruda, Adriano Sebollela

**Affiliations:** ^1^Department of Biochemistry and Immunology, Ribeirão Preto Medical School, University of São Paulo, Ribeirão Preto, Brazil; ^2^Center for Virus Research, Ribeirão Preto Medical School, University of São Paulo, Ribeirão Preto, Brazil; ^3^Department of Cell and Molecular Biology, Ribeirão Preto Medical School, University of São Paulo, Ribeirão Preto, Brazil; ^4^Department of Pathology and Forensic Medicine, Ribeirão Preto Medical School, University of São Paulo, Ribeirão Preto, Brazil; ^5^Department of Pharmacology, Ribeirão Preto Medical School, University of São Paulo, Ribeirão Preto, Brazil; ^6^Center for Research in Inflammatory Diseases (CRID), Ribeirão Preto Medical School, University of São Paulo, Ribeirão Preto, Brazil; ^7^Department of Physics and Chemistry, School of Pharmaceutical Sciences of Ribeirão Preto, University of São Paulo, Ribeirão Preto, Brazil; ^8^Department of Structural and Functional Biology (Anatomy), Institute of Biosciences, São Paulo State University, Botucatu, Brazil; ^9^Division of Neurosurgery, Department of Surgery and Anatomy, Ribeirão Preto Clinics Hospital, Ribeirão Preto Medical School, University of São Paulo, Ribeirão Preto, Brazil

**Keywords:** arboviruses, viral encephalitis, histocultures, neuroinflammation, neuroinfection, neurotropic virus, human brain

## Abstract

Oropouche virus (OROV) is an emerging arbovirus in South and Central Americas with high spreading potential. OROV infection has been associated with neurological complications and OROV genomic RNA has been detected in cerebrospinal fluid from patients, suggesting its neuroinvasive potential. Motivated by these findings, neurotropism and neuropathogenesis of OROV have been investigated *in vivo* in murine models, which do not fully recapitulate the complexity of the human brain. Here we have used slice cultures from adult human brains to investigate whether OROV is capable of infecting mature human neural cells in a context of preserved neural connections and brain cytoarchitecture. Our results demonstrate that human neural cells can be infected *ex vivo* by OROV and support the production of infectious viral particles. Moreover, OROV infection led to the release of the pro-inflammatory cytokine tumor necrosis factor-alpha (TNF-α) and diminished cell viability 48 h post-infection, indicating that OROV triggers an inflammatory response and tissue damage. Although OROV-positive neurons were observed, microglia were the most abundant central nervous system (CNS) cell type infected by OROV, suggesting that they play an important role in the response to CNS infection by OROV in the adult human brain. Importantly, we found no OROV-infected astrocytes. To the best of our knowledge, this is the first direct demonstration of OROV infection in human brain cells. Combined with previous data from murine models and case reports of OROV genome detection in cerebrospinal fluid from patients, our data shed light on OROV neuropathogenesis and help raising awareness about acute and possibly chronic consequences of OROV infection in the human brain.

## Introduction

Neurotropic viruses can potentially cause central nervous system (CNS) diseases (Abdullahi et al., 2020). The mechanisms that lead to neuroinvasion are not completely known for all neurotropic viruses, but some infection pathways include breach of the blood-brain barrier, Trojan horse effect, crossing of the choroid plexus, endothelial transcytosis, access by peripheral nerves and/or olfactory neurons (Koyuncu et al., 2013). Encephalitis, meningitis, myelitis and meningoencephalitis are clinical manifestations related to viral CNS infection, which include symptoms such as headache, photophobia, stiff neck, mental confusion, diplopia, seizures, and others. About 20–50% of diagnosed encephalitis are attributed to viruses, which underscores the importance of understanding virus infection in CNS (Tyler, 2018). Arthropod-borne viruses, also called arboviruses, from diverse viral families cause important human diseases around the world, and several of them are able to reach the CNS and cause neurological complications (Wasay et al., 2015). La Crosse virus (family *Peribuynaviridae*), for example, is a major agent of arboviral neuroinvasive disease in the United State, causing severe encephalitis (Ludlow et al., 2016). West Nile virus (family *Flaviviridae*), another important neurotropic arbovirus, may lead to significant cortical thinning in infected individuals, resulting in long-term neurological damage (Sejvar, 2007; Murray et al., 2018).

Oropouche virus (OROV) is an arbovirus of the Orthobunyavirus genus, *Peribuynaviridae* family, with important public health impact in several countries in South and Central Americas. In the last few years, it has been demonstrated that OROV is spreading for outside endemic regions, and as a consequence OROV has been pointed out as a potential epidemic candidate (de Souza Luna et al., 2017; Travassos Da Rosa et al., 2017; Romero-Alvarez and Escobar, 2018; Silva-Caso et al., 2019). OROV infection causes Oropouche fever disease, which the major symptoms are headache, myalgia, photophobia, and fever. In some patients, neurological symptoms have been reported, such as diplopia, nystagmus and meningoencephalitis (Sakkas et al., 2018; Sebastian Vernal et al., 2019). OROV has already been isolated from the cerebrospinal fluid of an infected individual (Pinheiro et al., 1982) and, more recently, the OROV genome has been detected in the cerebrospinal fluid from patients (Bastos et al., 2012; Sebastian Vernal et al., 2019).

Insights on OROV-neuroinfection have been provided by studies using murine models. Santos et al. (2014) showed that OROV can target the CNS in mice by the neural route and crossing the blood-brain-barrier. Moreover, OROV neuroinvasion seems to depend on the deficient activation of interferon-response factor 5, IRF5 (Proenca-modena et al., 2016). Once inside the rodent CNS, OROV infection results in glial activation and neuronal cell death (Rodrigues et al., 2011; Santos et al., 2012). It has been shown that OROV infects human leukocytes and monocytes, which could result in CNS invasion by the Trojan horse mechanism (de Souza Luna et al., 2017; Amorim et al., 2020). Despite these important findings of OROV neuroinfection, the human brain susceptibility to OROV infection has not yet been investigated in detail.

Despite the importance of rodent-based models in neurosciences, rodent and human brains have major biochemical and functional differences that limit the use of mouse or rat brain models in translational neuroscience (Miller et al., 2010; Hodge et al., 2019). To address this limitation, models using human cells have been developed as 2D cultures made with neural cell lineages, and 3D models, such as neurospheres and brain organoids. Although these models have provided important clues on CNS infection by neurotropic virus – in particular neurospheres and brain organoids that were recently used to investigate Zika virus and SARS-CoV-2 brain infection (Garcez et al., 2016; Wang, 2018; Jacob et al., 2020; Song et al., 2021) – none of them fully recapitulates the complexity of the mature human brain. In this context, human brain slice cultures prepared from surgically resected fresh tissue from adult patients has become a powerful alternative model in translational neuroscience (Jones et al., 2016). Slice cultures present the main advantage of preserving the architecture and neural connections found *in vivo* (Wellbourne-wood and Chatton, 2018; Qi et al., 2019). In recent years our group has developed a novel method to cultivate adult human brain tissue as slice cultures (Sebollela et al., 2012; Mendes et al., 2018; Fernandes et al., 2019). Notably, we have shown that cultured slices from adult human brains have preserved morphology, tissue viability, and neuronal function for at least 5 days *in vitro* (Mendes et al., 2018; Fernandes et al., 2019).

Most of the available studies of virus infection in human brain slices focused on the use of viruses as vectors for the expression of ectopic proteins (Andersson et al., 2016; Duigou et al., 2018; Ting et al., 2018). More recently, a few studies have been done focusing on understanding virus neuropathogenesis in fetal post-mortem human brain slice cultures (Martinez et al., 2011; Retallack et al., 2016). The outbreak of the neurotropic Zika virus has also stimulated the use of adult human brain tissue obtained from surgical resections in the investigation of adult CNS infection (Figueiredo et al., 2019). Here, motivated by the lack of studies on OROV neurotropism in humans, we have examined whether OROV can directly target human brain cells in a preserved tissue context, using an adult human brain slice culture model. We have seen that OROV infects microglia and neurons, and that this infection induces inflammatory and toxic responses, with TNF-α release and loss of cell viability. Moreover, this study also opens new possibilities on the use of adult human brain slice cultures to understand viral neuropathogenesis.

## Materials and Methods

### Virus Strain

Oropouche virus (strain BeAn 19991) was kindly provided by Prof. Luiz Tadeu Moraes Figueiredo (University of São Paulo, Ribeirão Preto Medical School, Brazil) and propagated in Vero cells to produce the virus stock. The titer of the virus stock was determined by cytopathic effect induction on Vero cells and expressed as 50% infectious dose (TCDI_50_). Conditioned medium from control Vero cells (mock) were used as negative control.

### Adult Human Brain Slice Cultures

Cultures were prepared as previously described (Mendes et al., 2018; Fernandes et al., 2019). Briefly, cortical tissue (anterior third of middle temporal gyrus) was obtained from patients submitted to an anterior temporal lobectomy with amygdalo-hippocampectomy for the treatment of refractory temporal lobe epilepsy. No lesion features were detected in both resonance exams and anatomopathological assessments. A fragment of cortex was collected at the surgical room immediately after resection and transported to the laboratory immersed in ice-cold oxygenated medium [50% v/v Hank’s balanced salt solution in Neurobasal A (Gibco)] supplemented with 10 mM Hepes and glucose 3 mg/ml. Tissue immersed in ice-cold oxygenated HBSS was sliced at 200 μm in a VT1000s automatic vibratome (Leica), and then transferred to 24-wells plate (one slice/well) with 600 μL of pre-warmed culture medium. The slice culture medium contains Neurobasal A (Gibco) medium supplemented with 1% Glutamax (Gibco), 1% Penicillin/Streptomycin (Gibco), 2% B27 (Gibco) and 0.25μg/mL Fungizona (Sigma). At day *in vitro* 1 (DIV1), 333μL were removed and 133μL of medium was added, and brain slices were cultivated in free-floating conditions until further treatments. One third of the culture medium was exchanged for fresh medium every 24 h. This procedure was approved by the Ethics Committee of the Ribeirão Preto Medical School (protocol HCRP #17578/15).

### Virus Infection in Adult Human Brain Slice Cultures

At days *in vitro* (DIV) 1 or 2, the medium was removed, and the slices were exposed to 3 × 10^6^ TCDI_50_ of OROV or an equivalent volume of mock medium. Infection was performed for 2 h at 37°C, 5% CO_2_. The inoculum was removed, the tissue was washed five times with PBS, and once with Neurobasal A medium (this medium was stored and referred to as 0 hours post-infection, hpi). The slices were maintained in fresh medium at 37°C and 5% CO_2_ until processing for subsequent analysis.

### Immunofluorescence

Brain slices (200 μm thick) were fixed in 4% paraformaldehyde for 24 h at 4°C, then incubated for 1 h with 0.3% Triton X-100 in PBS, washed with wash solution (0.2% Triton X-100 plus 1% BSA in PBS) and incubated with 0.1 M glycine for 1 h. The slices were blocked with 2% BSA for 2 h and incubated with the one of the following primary antibodies diluted in 0.2% Triton-X 100 and 2% BSA [mouse polyclonal anti-OROV (1:300; *in* house, provided by Prof. Luiz Tadeu Moraes Figueiredo), anti- Iba1 (1:1,000, Abcam, ab178846), anti-NeuN (1:1,000; Abcam; ab177487) or anti-GFAP (1:1,000; Merck, ab5804)] for 24 h at 4°C. Slices were washed three times with wash solution and incubated with one of the following secondary antibodies diluted in 2% BSA: Alexa Fluor 488 (Life Technologies; A-11001) or Alexa Fluor 568 (Life Technologies; A-11011). Finally, slices were stained with DAPI diluted in 2% BSA for 1 h, washed with wash solution, and incubated for 5 min with 70% ethanol followed by treatment with 0.1% Sudan Black (Merck) in 70% ethanol for 5 min. After washing (twice) with 70% ethanol, slices were mounted on slides coated with Fluoromont (Thermo Fisher). Stained tissues were analyzed on a Leica TCS SP5 laser scanning (Leica Microsystems) or Axio Observer LSM 780 (Carl Zeiss) confocal microscopes. Post-acquisition image processing and analyses were carried out using Image J/Fiji (Schindelin et al., 2012).

### Immunohistochemistry

Slices were fixed in 4% formaldehyde for 24 h at 4°C, and cryoprotect in 30% sucrose in PBS for 48 h. Slices were placed in a freezing microtome (Leica) at –40°C and cut at 30 μm. In 24-well plates containing 0.1 M PB solution (pH 7.4; Na_2_HPO_4_ Merck, 28 g; NaH_2_PO_4_.H_2_O Merck, 2.76 g, for 1 L), slices were incubated for 20 min with 10% hydrogen peroxide, washed and blocked with 2% donkey serum in 0.1 M PB for 40 min. Slices were immunolabeled with anti-NeuN antibody (1:1,000; Abcam; ab177487), overnight at 4°C. After washes, incubation with secondary antibody diluted in TBS-Tx 0.05 M was carried out for 2 h. Slices were then washed and incubated for 2 h with ABC Kit (Vector – Vectastain Standard Kit PK4000) diluted 1/200 according to the manufacturer’s instructions. Immunohistochemistry was performed using DAB + Nickel solution. Finally, slices were placed on frosted cut slides (Knittel), dehydrated, and covered with Permount (Fisher Scientific) and observed under a light microscope (Leica).

### TCID_50_ Assay

Virus particles in the supernatant from human brain slices were precipitated using polyethylene glycol (PEG) as described for other Bunyavirus (Hover et al., 2018), with minor modifications. Supernatants were incubated with 20% (PEG 6000, Sigma) diluted in 2.5 M NaCl (PEG/NaCl solution) in the proportion of 1: 4 (PEG/NaCl: supernatant). The mixture was gently homogenized, kept at 4°C for 1 h, and centrifuged at 10,800 *g* for 30 min at 4°C. The pellet was resuspended in 8 μL of PBS plus 2 μL of PEG, homogenized and kept at 4°C for 20 min, and centrifuged at 3,300 *g* for 30 min at 4°C. The pellet was resuspended in 50 μL of PBS, which was centrifuged at 11,600 *g* for 10 min at 4°C. The supernatant was immediately used to viral titration. Serial 10-fold dilutions of the concentrated supernatants were prepared in DMEM medium supplemented with 2% fetal bovine serum (FBS, Thermo Fisher) and added to Vero ATCC CCL81 cells monolayers plated on 96-well plates. After incubation at 37°C for 4 days, the titer was obtained by analyzing cytopathic effect events in each dilution and expressed as 50% infectious dose (TCID_50_) (Barbosa et al., 2018). The data was normalized by the mass of the slice correspondent to each supernatant.

### Transmission Electron Microscopy

Tissue was fixed with 3% glutaraldehyde in 0.1 M phosphate buffer for 24 h at 4°C and processed for electron microscopy as described below. Tissue was incubated with 2% osmium tetroxide by 2 h at 4°C, washed in 0.1 M phosphate buffer, and dehydrated in a graded ethanol series. The tissue was imbedded in resin (araldite 6005) and stained with toluidine blue to select the area of interest. The tissue was cut with a diamond knife and the contrast were obtained by incubating the tissue with 0.5% uranyl acetate and lead citrate. After careful drying of the grid, the material was analyzed using a JEOL JEM-100CX II electron microscope (JEOL, MA, United States) at the Multi-User Electronic Microscopy Laboratory of the Department of Cellular, Molecular Biology and Pathogenic Bioagents, Ribeirão Preto Medical School.

### Differentiation of Neuronal Cell Line and Oropouche Virus Infection

Differentiation of SH-SY5Y neuroblastoma cells was carried out as previously described (Lopes et al., 2019), with minor modifications. Cells were plated on coverslips previously coated with poly-D-lysine (Sigma) and maintained in Dulbecco’s Modified Eagle Medium (DMEM) containing 4,500 mg/L of glucose, L-glutamine (Gibco), 10% heat-inactivated fetal bovine serum (Gibco), 100 U/L of penicillin, and 0.1 g/L of streptomycin in a saturated humidity atmosphere, 5% CO_2_ at 37°C. After 24 h, the medium was replaced by differentiation medium containing 0.5% FBS, 100 U/L of penicillin and 0.1 g/L of streptomycin, and 10 μM of retinoic acid (RA; Sigma) in DMEM for 4 days. After this period, cells were incubated with differentiation medium supplemented with 50 ng/mL of brain-derived neurotrophic factor (BDNF, Sigma) for another 4 days. The differentiation medium was refreshed every 48 h. After differentiation, cells were incubated with the OROV inoculum for 1 h at 4°C (MOI = 1, multiplicity of infection) in DMEM supplemented with 2% FBS. The inoculum was removed, cell monolayer was washed and maintained in DMEM medium at 37°C and 5% CO_2_ until analysis.

### Cytokines Quantification

The levels of cytokines in the conditioned culture medium were determined by ELISA (BD OptiEIA, BD Biosciences). The results were expressed as picograms of each cytokine analyzed and normalized by the content of total protein in each slice (determined by the Bradford assay).

### Western Blot

Extracts from mock and infected slices were prepared in RIPA buffer (50 mM Tris, 150 mM NaCl, 1 mM EDTA, 1% Triton X-100 and 0.1% SDS, pH 7.5, supplemented with protease and phosphatase inhibitor) using a motorized pestle. Homogenates were centrifuged at 4°C for 15 min at 16,000 × *g* and supernatants collected. Protein concentration was determined using the Bradford assay. Aliquots containing 50 μg total protein were resolved on Mini-PROTEAN TGX^TM^ Precast Protein Gels, 4–15% (Biorad). The proteins were transferred to nitrocellulose membranes using a Transblot turbo apparatus (Biorad). Membranes were blocked at 25°C for 1 h with 2.5% BSA in TBS-T (0.05 M Tris, 0.15 M NaCl, 0.1% Tween 20, pH 7.5) and incubated overnight with anti-HLA-DR (1:1,000, Abcam, ab92511), anti-Tau phospho S396 [E178] (1:1,000, Abcam, ab32057), or anti-β-actin (1:10,000, Merck, MAB1501). Primary antibodies were diluted in TBS-T containing 2.5% BSA. After washing and incubation with HRP-conjugated secondary antibodies (GE Healthcare), membranes were developed using ECL-Prime (GE Healthcare). Quantitative analyses were made using Image J/Fiji.

### MTT Assay

Cell viability in the cultured slices was assessed using the MTT assay as in Mendes et al. (2018) and Fernandes et al. (2019). Briefly, slices were incubated with 0.5 mg/ml MTT for 3 h at 37°C, washed, and homogenized in isopropanol/0.01 N HCl. The homogenate was centrifuged at 5,000 rpm for 2 min and the supernatants were collected for quantification of O.D. at 540 nm. The values were normalized by the mass of each slice.

### Statistical Analysis

Statistical analysis was performed using Graph Pad Prism 6 (Graph Pad Software). Means were compared using Student’s *t*-test (for two groups) or One-Way ANOVA (for three groups), as indicated in the legends for the figures. The number of tissue donors included in each experiment has been referred to as “*n*” throughout the text.

## Results

### Adult Human Brain Cells Are Susceptible to *ex vivo* Infection by Oropouche Virus

Previous studies demonstrated neuroinfection by OROV in rodent models (Rodrigues et al., 2011; Santos et al., 2012, 2014; Proenca-modena et al., 2016). On the other hand, no conclusive information about OROV infection in human neural cells is yet available. To address this, we used a novel human brain slice culture model developed by our group (Mendes et al., 2018; Fernandes et al., 2019). The slices are prepared from tissue collected at the surgical room, from adult patients undergoing surgical resection of epileptic foci. In our previous works, we have observed no major constraints in cell viability and tissue morphology/cytoarchitecture in these human brain-derived slice cultures that could impose a significant limitation to the use of this model for studying virus neuroinfection (Mendes et al., 2018; Fernandes et al., 2019). The tissue fragment used to prepare the slice cultures correspond to the temporal cortex resected to provide access to the hippocampal epileptogenic area. A significant advantage of this model is to preserve the original cellular population and connections found in the adult human brain, as we have previously shown the presence of neurons, microglia and astrocytes by light microscopy in re-sectioned slices submitted to DAB-Nickel immunostaining (Fernandes et al., 2019).

**FIGURE 1:**
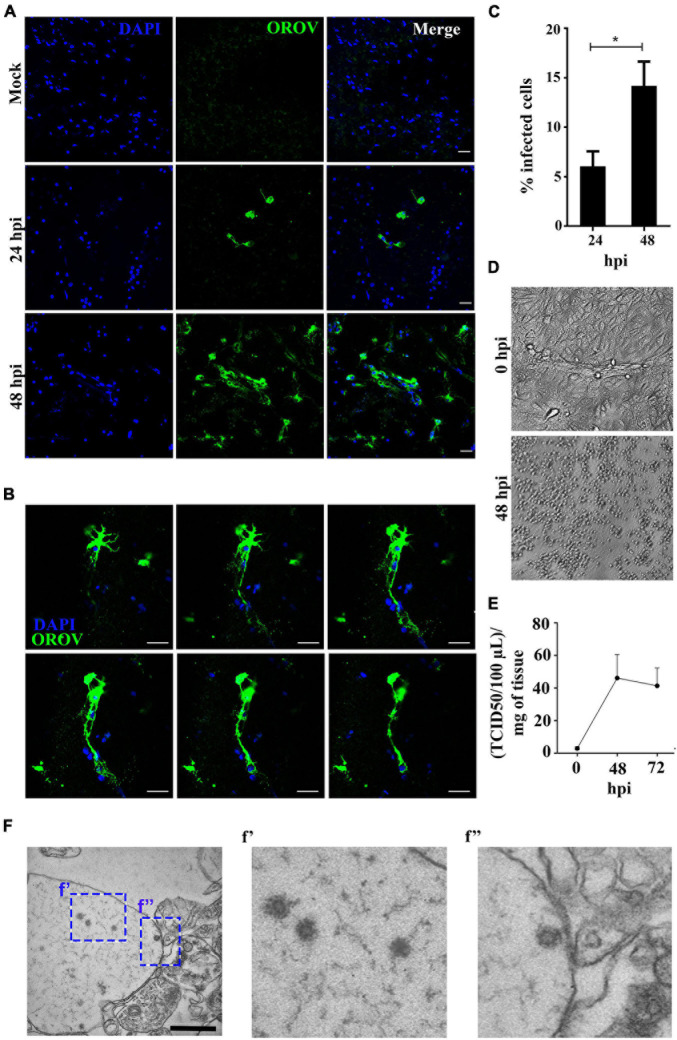
Human neural cells in adult human brain slices are susceptible to *ex vivo* infection by Oropouche virus (OROV). **(A)** Representative confocal images of uninfected control (mock) and OROV-infected slices at 24 and 48 hours post infection (hpi). Tissue sections were stained for OROV (green) and nuclei (DAPI, blue) (Scale bar = 20 μm). **(B)** Representative sequential Z-stack images from a cell infected by OROV in a human brain slice. Tissue was labeled for nuclei (DAPI, blue) and OROV antigen (green) (Z-Stacks = 0.5 μm) **(C)**. Quantification of OROV positive cells at 24- and 48 hpi (*n* = 4–9 donors). **(D)** Representative cytopathic effect observed in Vero cells inoculated with the 0- or 48 hpi supernatant (10^3^ dilution) from the one brain slice culture. **(E)** Virus titers in the supernatant from brain slice infected by OROV (*n* = 2 donors). **(F)** Representative transmission electron microscopy images of a human brain slice 48 hpi. Structures with typical virus particle morphology were observed (**f’** and **f”**, highlighted by the dashed square) (Scale bar = 1 μm). Results are show as the mean ± SD. (*P < 0.05; Student *t*-test).

To evaluate if human neural cells in a preserved tissue architecture are susceptible to OROV infection, we have exposed human brain slices in culture to OROV for either 24- or 48-h, and evaluated the presence of viral antigens in the cells using a polyclonal anti-OROV antibody (Rodrigues et al., 2011). We detected viral antigens in human neural cells at both time points tested, with a significant increase in the number of infected cells at 48 hours post infection (hpi) compared to 24 hpi (Figures 1A–C). Sequential images of cells positive for OROV in human brain slices showed virus antigens throughout the cell cytoplasm (Figure 1B). To investigate if human neural cells were permissive for the production of novel virus particles, we collected supernatants from OROV-infected slices and performed OROV titration in OROV-susceptible Vero cell monolayers by TCID_50_. OROV progeny accumulated about 40-fold in supernatant of OROV-infected human brain slices at 48 hpi as compared to time zero, and had only a modest decrease at 72 hpi (Figures 1D,E). The supernatant collected at 24 hpi was not titrated because only a few cells were positive for OROV antigen by immunostaining at that time point (Figure 1). The presence of OROV virus particles in infected human brain slices was also observed by transmission electron microscopy at 48 hpi, which showed structures with typical morphology and diameter expected for family *Peribunyaviridae* (Talmon et al., 1987; Travassos Da Rosa et al., 2017; Figure 1F). These structures were not observed in mock-infected slices (data not shown). In conjunction, these data indicate that neural cells in adult human brain tissue are indeed susceptible to OROV.

### Identification of Neural Cell Types Infected by Oropouche Virus in Adult Human Brain Slices

**FIGURE 2:**
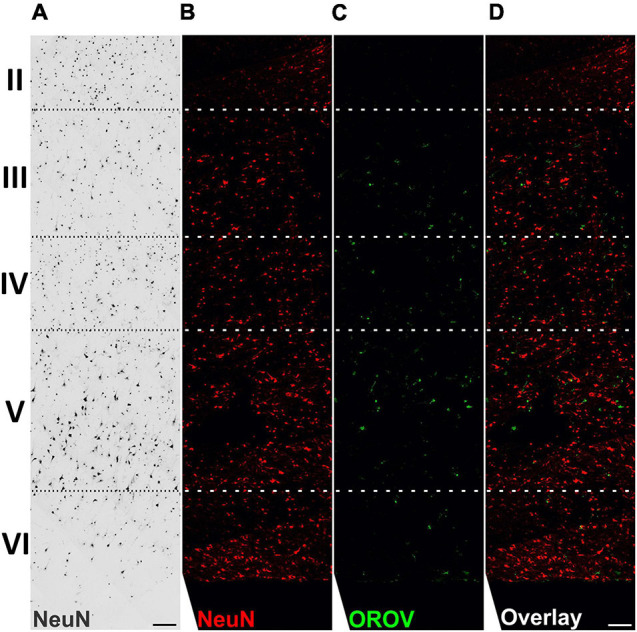
Oropouche virus infection in adult human brain slices is distributed along deep cortical layers. Adult human brain slices in culture (day *in vitro* 4) from middle temporal gyrus were labeled with the neuronal marker NeuN, and with anti-OROV antibody **(C,D)**. Representative multiphoton images from an OROV-infected slice **(B–D)** are shown. A brightfield image from a control (non-infected) slice is also shown for reference **(A)**. Cortical brain layers (II–VI), assigned according to neuronal morphology and density, are indicated on the left. Scale bar = 100 μm.

**FIGURE 3:**
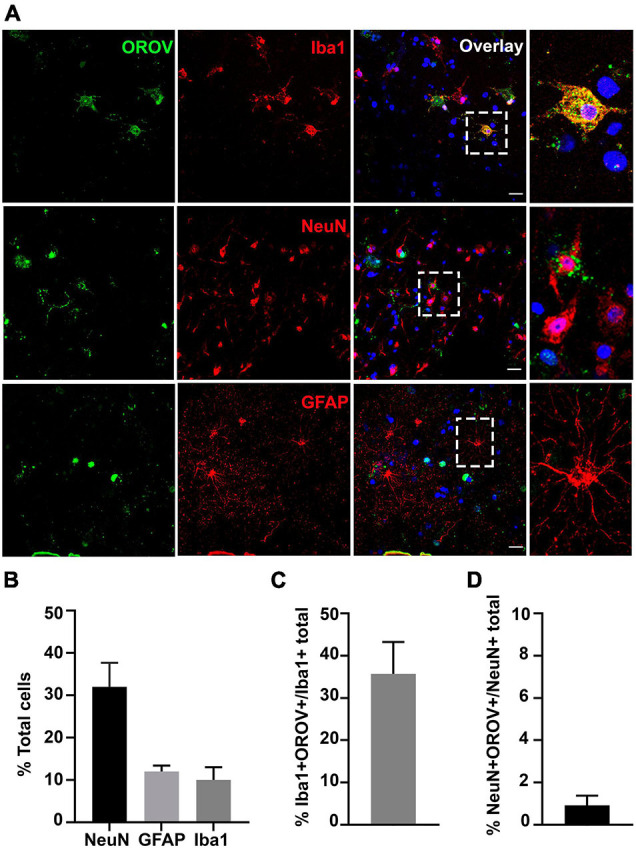
Oropouche virus targets microglia and neurons in adult human brain slices. **(A)** Representative confocal images of immunofluorescence labeling for OROV (green) and Iba1, NeuN or GFAP (red) in cultured slices 48 hpi. Scale bar = 20 μm. **(B)** Percentages of Iba1, NeuN and GFAP positive cells among total cells. **(C,D)** Percentage of OROV-positive cells among cells positive for Iba1 or NeuN, respectively. Results are shown as mean ± SD (*n* = 2–5 donors).

To gain information on the pattern of OROV infection in neocortical areas, we evaluated virus antigens distribution along cortical layers in OROV-infected brain slices. Although some care must be taken when analyzing cortical tissue obtained from pharmaco-resistant epileptic patients, as it is known that in some cases neurons can present abnormal orientations (Al Sufiani and Ang, 2012), in our previous work we have been able to clearly identify all neuronal cortical layers in similar slice cultures (Fernandes et al., 2019). Here, we have seen that OROV infection is present in most of the cortical layers identified, being more readily detected in deeper layers (Figure 2). In order to determine which neural cell types are infected by OROV in human brain slices in culture, we performed double immunostainings to detect the viral antigens and one of the following neural cell markers: Iba1 for microglia; NeuN for neurons; or GFAP for astrocytes. We observed that at 48 hpi both microglial cells and neurons were positive for OROV antigen, while no infected astrocytes were detected (Figure 3A). Despite the fact that microglia represent 10% of the total cells present in slices (Figure 3B), approximately 35% of these cells were positive for OROV antigens (Figure 3C). On the other hand, neurons represent 33% of total cells (Figure 3B), but only approximately 2% of them were OROV-positive (Figure 3D). Astrocytes correspond to 11% of total cells (Figure 3B), but although being a significant cell population in these slices, a careful search (11 fields, including 0.5 μm z stacks), indicated a lack of co-localization between OROV proteins and GFAP positive cells in adult human brain slices. In conjunction, these quantifications indicate that microglial cells are preferentially infected by OROV in adult human brain cortex.

**FIGURE 4:**
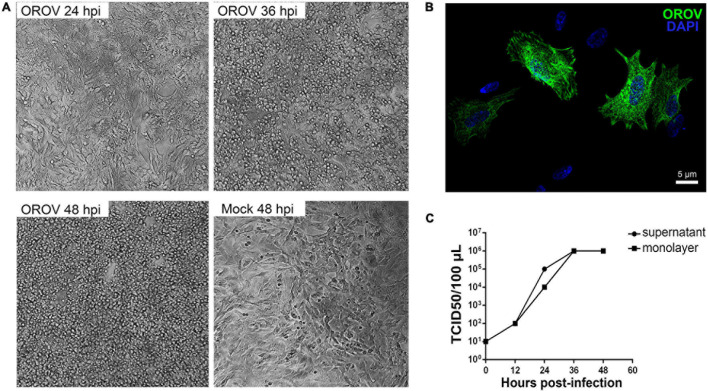
Differentiated human neuroblastoma cells support OROV replication. Neuroblastoma SH-SY5Y cells were differentiated into mature neurons and inoculated with OROV. **(A)** Cytopathic effect in SH-SY5Y cells was observed 24, 36, and 48 hpi. Images were obtained using the 20× objective. **(B)** Representative immunofluorescence labeling of OROV antigen (green) in SH-SY5Y cells exposed to OROV. **(C)** Virus titers in supernatants and monolayer at different times post infection of cultures of SH-SY5Y cell line (*n* = 3 biological replicates).

To further investigate the OROV potential to infect human neurons, we induced differentiation of the human neuroblastoma cell line SH-SY5Y into a mature neuronal phenotype and exposed these cultures to OROV. We observed intense cytopathic effect at 24-, 36-, and 48 hpi (Figure 4A), and infection was confirmed by the detection of strong OROV antigens by immunostaining (Figure 4B). In addition, infectious OROV particle production by infected differentiated SH-SY5Y cells was confirmed by TCID_50_ assay in supernatants and remaining cell homogenates, which was time-dependent and reached a plateau at 48 h post infection (Figure 4C), roughly in agreement with the time when most cells presented cytopathic effect. These data reinforced the notion that OROV can infect human neurons, even though the degree of neuronal infection by OROV in the context of preserved human brain tissue seemed to be significantly less abundant than that of microglia.

### Adult Human Brain Tissue Response to Oropouche Virus Infection

**FIGURE 5:**
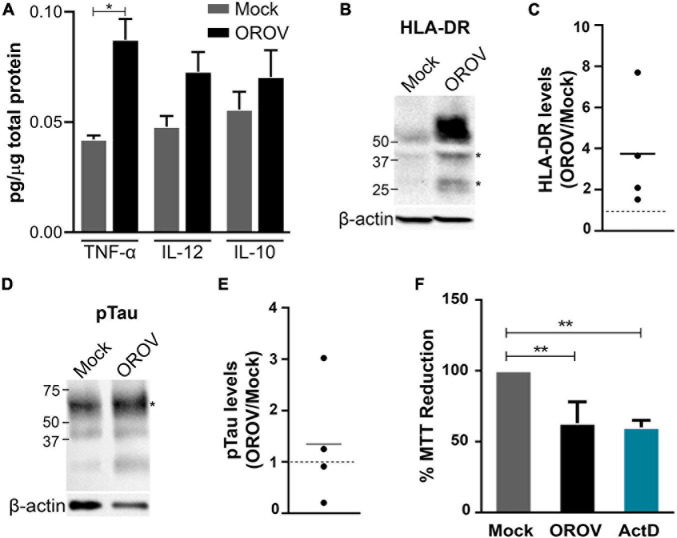
Oropouche virus infection induces an inflammatory and toxic response in adult human brain slices. **(A)** Cytokine levels in supernatants of brain slice cultures (*n* = 3 donors; *P < 0.05; Student *t*-test). **(B,D)** Representative immunoblots probing HLA-DR or phosphorylated Tau (pTau), respectively. Bands used for quantification are indicated with asterisks (*). β-actin was used for normalization. **(C,E)** HLA-DR/β-actin and pTau/β-actin ratios in four independent infected slices. Mock levels are indicated by dotted lines (*n* = 4 donors). **(F)** Cell viability in slices determined by the MTT assay 48 hpi. Actinomycin D (ActD) was used as positive control for cell death (*n* = 3 donors; **P < 0.001; One Way ANOVA).

The demonstration of OROV infection of both microglia and neurons in human brain slices raised the possibility of augmented release of inflammatory signals by these cells in response to OROV infection. We therefore determined the levels of cytokines in the conditioned medium of OROV-infected human brain slices by ELISA. Elevated levels of cytokines have been described as an important initial response to experimental virus infection, including against OROV (Rempel et al., 2005; Kawanokuchi et al., 2006; Amorim et al., 2020). Interestingly, we observed a significant increase in TNF-α release by brain slices infected with OROV, as compared to control slices (Figure 5A). On the other hand, no significant differences were found in IL-12 or IL-10 levels between mock and OROV-infected human brain slices (Figure 5A). We also detected an increase in TNF-α mRNA levels in OROV-infected brain slices in samples from a single donor (Supplementary Figure 1). This inflammatory response seems to be not associated to tissue processing prior culturing, since we have observed a clear change in microglial morphology, including increased cell body area and reduced amount and length of branches, known features of activated microglia (Tay et al., 2017; Koss et al., 2019; Tan et al., 2020), after a pro-inflammatory stimulus with LPS at DIV2 (Supplementary Figure 2), which indicates microglia was not highly activated at the time of experimental infection with OROV.

Since microglia has been described as the main source of TNF-α in the brain (Riazi et al., 2008; Wang et al., 2015), we further investigated microglial activation status in OROV infected-brain slices. Firstly, we have evaluated the expression of iNOS, a functional marker of M1 (pro-inflammatory) microglia phenotype (Lisi et al., 2017). In line with the notion of microglial activation upon OROV infection, we have observed an increase in iNOS mRNA levels in samples from a single donor (Supplementary Figure 1). Moreover, we evaluated the protein levels of a classical activation marker for microglial cells, HLA-DR, a MHC class II cell surface receptor known to play a role in virus clearance (Walker and Lue, 2015; Hendrickx et al., 2017; Klein et al., 2019; Chhatbar and Prinz, 2021). Remarkably, we found an augment in HLA-DR levels in all OROV-infected slices tested (Figures 5B,C). In addition to elevated iNOS and TNF-a mRNA levels, and HLA-DR and TNF-α proteins in OROV-infected human brain slices, we also observed microglia with apparent amoeboid morphology near infected cells, suggesting microglial activation (Supplementary Figure 3).

Finally, to investigated the impact of OROV infection on neuronal viability, we evaluated Tau phosphorylation (Serine396) levels in OROV-exposed adult human brain slices, as increased pTau (Ser396) has been shown to be a solid marker of neurodegeneration (Wang et al., 2013). We observed an increase in pTau (ser396) levels in 2 out of 4 slices tested, indicating neuronal damage in part of the infected slices (Figures 5D,E). Furthermore, OROV-infection led to a reduction in tissue viability, as determined by the MTT assay (Figure 5F). This reduction in viability was not associated with a reduction in mitochondrial content (Supplementary Figure 4), suggesting that it corresponds to cell death. Collectively, these data indicated that OROV infection triggers a microglia-mediated inflammatory response and neurotoxicity in adult human brain slices.

## Discussion

Previous reports indicate that OROV can reach the human CNS (Pinheiro et al., 1982; Bastos et al., 2012; Sebastian Vernal et al., 2019). In the present study, we have used human brain slice cultures, a powerful model in investigations on neural connections and cytoarchitecture typical of the adult human brain, to address the question about human brain susceptibility to OROV infection. We have shown that OROV is capable of infecting human neural cells, namely microglia and neurons, with preponderance of microglia infection. Furthermore, we have shown that OROV infection leads to an inflammatory response with increased TNF-α release, microglia activation markers, neuronal damage and loss of tissue viability.

We detected OROV antigen inside neural cells, which indicate virus protein translation. We also detected infectious virus particles produced by neural cells, and virus structures inside some of these cells, indicating that infection in neural cells is productive. This productive infection in human brain slices could result in OROV spread throughout the tissue, which could result in brain damage. The production of virus proteins *per se* can also contribute to brain damage. For instance, it is known that NSs, a non-structural protein that is the major virulence factor of Schmallenberg virus, an Orthobunyavirus, can cause shut-off of the host cell protein synthesis and consequent cell dysfunction (Kraatz et al., 2018). In HIV-1 neuroinfection, both the release of virus particles, viral proteins and cytokines contribute to neurological changes, with synaptodendritic damage and neurodegeneration (dos Reis et al., 2020). Some patients infected by West Nile virus, an important neurotropic arbovirus, can present symptoms like depression, memory loss, and motor dysfunction as chronic sequelae (Fulton et al., 2020). Therefore, the OROV-proteins production and infectious virus particles spread may result in tissue damage and perhaps to chronic consequences.

The differences between murine and human brain cells, including, but not limited to, the structure and expression levels of cell receptors, may significantly impact virus infection and consequently cell susceptibility (Hodge et al., 2019). SARS-CoV 1 and 2, for example, depends on the interaction with human-specific receptors for efficient infection, which motivated the development of transgenic mouse expressing the human receptor for studies on SARS-CoV disease (Li et al., 2004; Winkler et al., 2020; Kumari et al., 2021). A characterization of OROV infection in brain tissue has been presented in studies using hamster and mouse models, and revealed a higher neuronal susceptibility to infection (Rodrigues et al., 2011; Santos et al., 2012). In the adult human brain slice model, OROV infection was also observed in neurons, although microglia were the preferential targeted cell. Interestingly, in our transmission electron microscopy data the structures resembling OROV particles were observed next to synaptic terminals. In addition, using differentiated SH-SY5Y neuroblastoma cells we showed the capacity of OROV to infect and replicate in human neurons. We also observed that *in vitro* OROV infection induces cytopathic effects in these cells. We also observed an increase in pTau (ser396) levels in 2 out of 4 human brain slices tested. This distinct profile in Tau phosphorylation may be attributed to intrinsic differences of tissue obtained from multiple donors, that could lead to different cellular responses. This neuronal damage can be either directly related to OROV infection – since OROV induced cytopathic effects in a neuronal lineage – or to be a consequence of the release of inflammatory molecules from microglia in response to OROV infection. A combination of both mechanisms, or the contribution of other sources of inflammatory/toxic molecules cannot be ruled out at this point. These data indicate that OROV has the potential to cause neuronal damage, which could lead to neural networking dysfunctions and consequent neurological symptoms.

The high frequency of microglial cells positive for OROV antigen in the present study indicates that OROV infection may cause important dysfunction. Microglial cells are considered tissue-resident macrophage-like innate immune cells of the CNS, and the main defense of the human brain against several CNS pathogens (Chatterjee et al., 2013). Furthermore, microglia has an important role in controlling the spread of neurotropic viruses inside the CNS (Fekete et al., 2018; Chen et al., 2019). It is also important to note, however, that microglia infection may function as a reservoir of virus in the brain (Wallet et al., 2019). The observed preference of OROV to infect microglia could guide experimental identification of the host cell receptor responsible for OROV infection in the human brain, starting from *in silico* searches of putative host cell receptors guided by the microglia proteome. Studying ligand-receptor relation in OROV neural infection is needed, since few studies on OROV, a neglected virus, and its cell receptor are available.

Oropouche virus-infected human brain slices release increased levels of TNF-α. Furthermore, in the CNS TNF-α is released mainly by microglia, although it can also be secreted from other types of brain cells (Riazi et al., 2008; Wang et al., 2015). We have also observed increased HLA-DR protein levels and iNOS expression, both markers of microglial activation (Walker and Lue, 2015; Hendrickx et al., 2017; Lisi et al., 2017; Klein et al., 2019; Chhatbar and Prinz, 2021). We have also observed microglial amoeboid phenotype in OROV-infected human brain slices. The amoeboid phenotype is an evidence of the presence of activated microglia (Fernández-Arjona et al., 2019), which, together with TNF release, increased HLA-DR protein levels and iNOS expression suggest that the ability to react as part of an innate immune response is important in OROV infection is preserved in the human brain slice culture model. This process has also been observed in other arbovirus infection model, using murine spinal cord slices (Quick et al., 2014, 2017). Although it has been shown that arbovirus infections in immunocompetent patients do not lead to significantly elevated levels of cytokines in the cerebrospinal fluid (Bastos et al., 2015), OROV-neuroinvasion has been pointed out as a major pathogenesis consequences in immunodeficient animal models (Proenca-modena et al., 2016). After the neuroinvasion, TNF release could play an important role in the control of OROV spread into the CNS. It is important to note, however, that although the inflammatory response could be critical to virus control, it can also contribute to tissue damage and neurological consequences, as observed in HSV-1 encephalitis in adults (Bradshaw and Venkatesan, 2016).

The present study also revealed that OROV infection induces cell death in human brain slices, The results on mitochondria content, probed using the citrate synthase assay, indicated a lack of difference between OROV- and mock-infected brain slices, supporting the interpretation that loss in tissue viability, measured using the MTT assay, indeed resulted from cell death. In line with our findings, previous studies reported cell death in the brain induced by OROV infection in mice (Santos et al., 2012; Proenca-modena et al., 2016). OROV infection also caused apoptosis in HeLa cells (Acrani et al., 2010). Cell death has also been observed in spinal cord slices upon experimental infection with other arbovirus (Clarke et al., 2014). Although the mechanisms associated to OROV-induced loss of viability in human brain slices need to be further investigated, based on these previous findings, and on our present demonstration of augmented release of TNF-α, iNOS expression and HLA-DR protein levels it is possible that neuronal damage, cell death and neuroinflammation are behind neuropathogenesis in a part of OROV patients.

Oropouche virus genome detection in the CNS and the neurological symptoms and signs found in some patients raise the possibility that OROV can infect brain cells. In the present work, we show for the first time that OROV can indeed infect human neural cells, namely microglia and neurons, in an *ex vivo* model that preserves brain cytoarchitecture and connections. This infection resulted in the release of an inflammatory cytokine and cell death, which *in vivo* could have neurological consequences. Our findings support the notion of OROV neurotropism, and raise a concern about either acute or chronic consequences of OROV infection to the adult human brain. Furthermore, it opens the possibility of using the free-floating human brain slices in studies on the neuropathology and therapeutic interventions for neurotropic virus infections.

## Data Availability Statement

The original contributions presented in the study are included in the article/Supplementary Material, further inquiries can be directed to the corresponding authors.

## Ethics Statement

The studies involving human participants were reviewed and approved by Ribeirão Preto Clinics Hospital and Ribeirão Preto Medical School Research Ethics Committee, at the University of São Paulo (USP). The patients/participants provided their written informed consent to participate in this study.

## Author Contributions

GA, EA, and AS contributed to the study design and interpreted the results of the experiments. GA, JS, NM, MP, GN, NP, IP, FV, and GF performed the experiments. GA, JS, NM, GN, RC, and JH-J analyzed the data. GP-G contributed to the collection of tissue from patients. LN, TC, JH-J, FC, and LA contributed to the discussion of the experiments. GA drafted the manuscript. EA and AS revised the manuscript. All the authors contributed to the article and approved the final version.

## Conflict of Interest

The authors declare that the research was conducted in the absence of any commercial or financial relationships that could be construed as a potential conflict of interest.

## Publisher’s Note

All claims expressed in this article are solely those of the authors and do not necessarily represent those of their affiliated organizations, or those of the publisher, the editors and the reviewers. Any product that may be evaluated in this article, or claim that may be made by its manufacturer, is not guaranteed or endorsed by the publisher.
