# Supplementary material for: Neural Infection by Oropouche Virus in Adult Human Brain Slices Induces an Inflammatory and Toxic Response
Source: Front Neurosci. 2021 Nov 23;15:674576. doi: 10.3389/fnins.2021.674576 (PMC8651276; doi:10.3389/fnins.2021.674576)
Supplement: Supplementary file 1 [file Data_Sheet_1.pdf]

Supplementary Material

MATERIAL AND METHODS

RNA extraction and RT-qPCR. Total RNA was extracted from one slice per group (Mock and OROV), using Trizol® (Invitrogen), according to manufacturer's instructions, and quantified using NanoDrop One/One c (ThermoFisher Scientific). All samples presented 260/280nm and 230/260nm ratios between 1.8 and 2.2. For each sample, 200 ng of total RNA was used for reverse transcription and cDNA synthesis. The reactions were performed using High-Capacity cDNA Reverse Transcription Kit (Applied Biosystems), according to manufacturer's instructions. Gene expression was determined through qPCR using StepOne TM Real-Time PCR System (Applied Biosystems) and SYBR® Fast qPCR Kit Master Mix (Kapa Biosystems). PCR amplification was performed without the extension step (95°C for 3 minutes, followed by 40 cycles of 95°C for 3 seconds and 60°C for 20 seconds). Fluorescence levels were measured during the last step of each cycle (60°C). In all reactions, a negative control, with no sample, was tested. The relative quantities of transcripts were calculated by the delta-delta Ct method (Vandesompele et al., 2002).

Treatment with LPS and immunostaining to Iba1. At DIV1, the volume of culture medium was reduced to 200 µl and 1 µg/ml of LPS or the same volume of PBS (control) was added. After 15 minutes, the slices were washed with Neurobasal A medium and fresh, supplemented culture medium was added. After 24h, the slices were collected for immunohistochemistry, performed as previously described (Mendes et al, 2018). Slices were fixed in buffered paraformaldehyde (4%) overnight at 4 °C, cryoprotected with 30% sucrose, frozen and cut at 30 µm in a freezing microtome (Leica). Sections were transferred to 24-well plates for free-floating immunostaining. Briefly, sections were incubated for 40 min in 2% normal donkey serum in phosphate buffer and incubated overnight with anti- Iba1 (Abcam, ab178846; 1:2000 dilution). After incubation with avidin-biotin secondary antibody (Vectastain, Kit Standard, PK-4000, Vector), immunoreactivity was revealed using DAB+0.04% nickel ammonium. Finally, sections were mounted on gelatin coated slides, air dried, dehydrated in increasing ethanol solutions, delipified in xylene, and imaged in a BX 61 microscope (Olympus) with 40x magnification lens.

Citrate synthase activity assay. The slices were homogenized in triethanolamine-HCl buffer 0.1 M (pH 8.0), 0.3 mM acetyl-CoA, 0.5 mM oxaloacetate, 0.25% Triton X-100 and 0.1 mM 5,5'-dithiobis-2-nitrobenzoic acid (DTNB) (Catalão et al., 2017). The homogenates were centrifuged at 12000xg at 4 °C for 10 min and the supernatant was collected (Spinazzi et al., 2012). The protein concentration was determined by Bradford assay. The reaction was started by the addition of 40µg of protein. Citrate synthase activity was determined spectrophotometrically according to the method of (Srere, 1969).

TABLE

Table 1. Demographic data for donors of tissue included in this study

Patient	Gender	Age (Years)
1	Female	50
2	Female	44
3	Male	51
4	Male	26
5	Female	37
6	Male	36
7	Female	41
8	Male	30
9	Male	40
10	Female	51
11	Male	44
12	Female	30
13	Female	56
14	Male	25
15	Female	42
16	Female	38
17	Female	32

Table 2. Primer sequences for qPCR and amplicon sizes.
Reference gene (GAPDH) and target genes (iNOS and TNF alpha).

Target	Sequence 5'-3'	Amplicon (pb)
**iNOS**	F- GTTCTCAAGGCACAGGTCTC	127
	R- GCAGGTCACTTATGTCACTTAT	
**TNF-α**	F- CTCTTCTGCCTGCTGCACTTTG	135
	R- ATGGGCTACAGGCTTGTCACTC	
**GAPDH**	F-GTCTCCTCTGACTTCAACAGCG	131
	R-ACCACCCTGTTGCTGTAGCCAA	

FIGURES

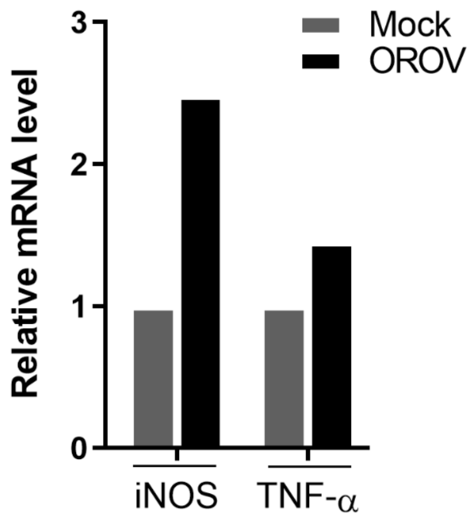

Figure S1. OROV infection elevates mRNA levels of the pro-inflammatory molecules iNOS and TNF-α. GAPDH expression was used for normalization. (n = 1 donor).

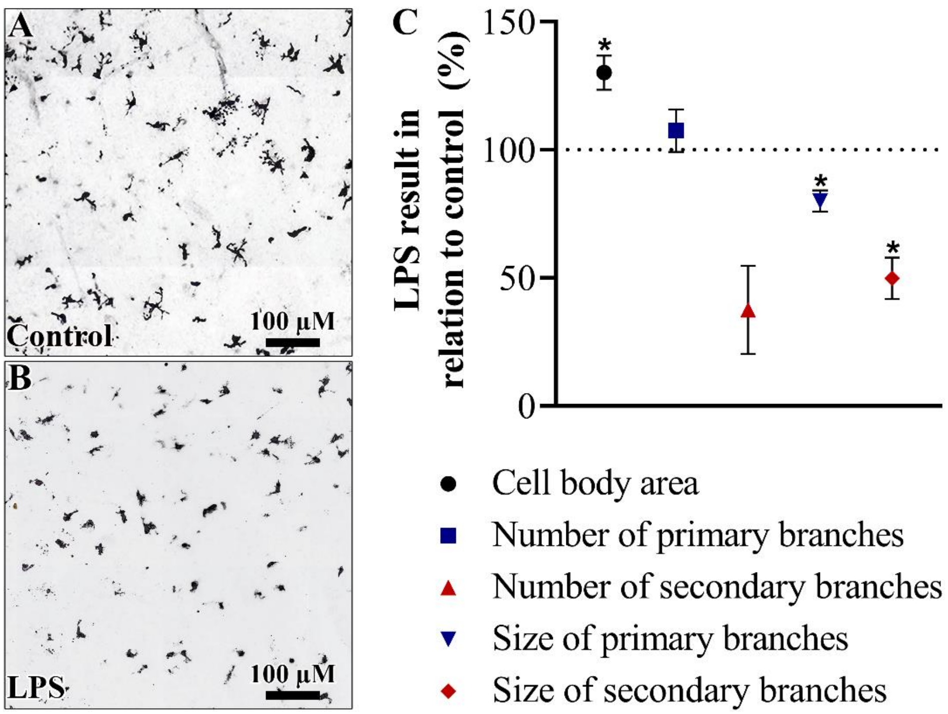

Figure S2. Microglial morphology changes induced by LPS treatment. Slices at DIV 2 were treated with PBS **(A)** or 1 µg/ml LPS **(B)** 24h before immunohistochemistry against Iba-1. **(C)** Microglia morphology parameters in slices treated with LPS in relation to control (represented by the dotted line). N = 60 cells per condition. $*$ $p < 0.05$ compared to control (Student's T test).

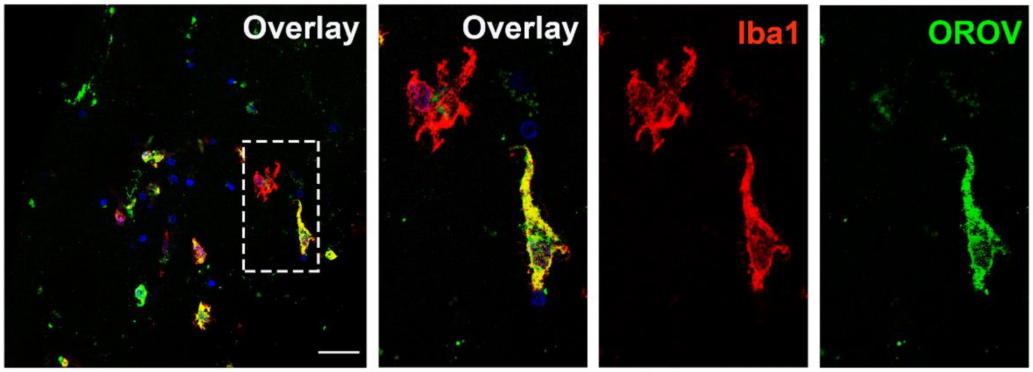

Figure S3. Representative image of microglia in human brain slice infected ex vivo with OROV. Tissue was labeled with viral antigen (green) and Iba-1 (red). Dashed circles are non-infected microglia with characteristic activated morphology (ameboid cell) near an infected cell. Scale bar = 20 µm.

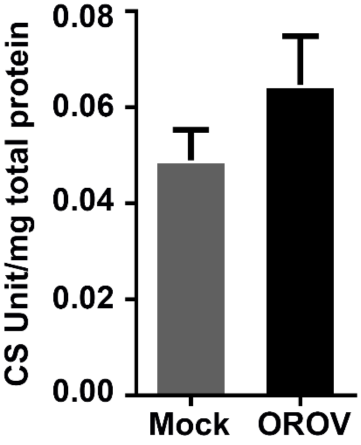

Figure S4. Mitochondria content was determined by citrate synthase (CS) activity. There was no significant difference between infected and non-infected cells (Student t-test, n=4).

REFERENCES

Catalão, C. H. R., Santos-Júnior, N. N., da Costa, L. H. A., Souza, A. O., Alberici, L. C., and Rocha, M. J. A. (2017). Brain Oxidative Stress During Experimental Sepsis Is Attenuated by Simvastatin Administration. *Mol. Neurobiol.* 54, 7008–7018. doi:10.1007/s12035-016-0218-3.

Spinazzi, M., Casarin, A., Pertegato, V., Salviati, L., and Angelini, C. (2012). Assessment of mitochondrial respiratory chain enzymatic activities on tissues and cultured cells. *Nat. Protoc.* 7, 1235–1246. doi:10.1038/nprot.2012.058.

Srere, P. A. (1969). [1] Citrate synthase. [EC 4.1.3.7. Citrate oxaloacetate-lyase (CoA-acetylating)]. *Methods Enzymol.* 13, 3–11. doi:10.1016/0076-6879(69)13005-0.

Vandesompele, J., De Preter, K., Pattyn, F., Poppe, B., Van Roy, N., De Paepe, A., et al. (2002). Accurate normalization of real-time quantitative RT-PCR data by geometric averaging of multiple internal control genes. *Genome Biology 2002 3:7* 3, 1–12. doi:10.1186/GB-2002-3-7-RESEARCH0034.
